# Human Platelet Lysate as a Functional Substitute for Fetal Bovine Serum in the Culture of Human Adipose Derived Stromal/Stem Cells

**DOI:** 10.3390/cells8070724

**Published:** 2019-07-15

**Authors:** Mathew Cowper, Trivia Frazier, Xiying Wu, J. Lowry Curley, Michelle H. Ma, Omair A. Mohiuddin, Marilyn Dietrich, Michelle McCarthy, Joanna Bukowska, Jeffrey M. Gimble

**Affiliations:** 1 School of Medicine, Tulane University, New Orleans, LA 70112, USA; 2 LaCell LLC, New Orleans, LA 70148, USA; 3 Obatala Sciences Inc., New Orleans, LA 70148, USA; 4 Axosim Inc., New Orleans, LA 70112, USA; 5 Louisiana State University School of Veterinary Medicine, Baton Rouge, LA 70803, USA; 6 Institute for Animal Reproduction and Food Research, Polish Academy of Science, 10-748 Olsztyn, Poland

**Keywords:** adipogenesis, adipose-derived stromal/stem cells, chondrogenesis, colony forming unit-fibroblast, fetal bovine serum, human platelet lysate, mesenchymal stem cell, osteogenesis, regenerative medicine

## Abstract

Introduction: Adipose derived stromal/stem cells (ASCs) hold potential as cell therapeutics for a wide range of disease states; however, many expansion protocols rely on the use of fetal bovine serum (FBS) as a cell culture nutrient supplement. The current study explores the substitution of lysates from expired human platelets (HPLs) as an FBS substitute. Methods: Expired human platelets from an authorized blood center were lysed by freeze/thawing and used to examine human ASCs with respect to proliferation using hematocytometer cell counts, colony forming unit-fibroblast (CFU-F) frequency, surface immunophenotype by flow cytometry, and tri-lineage (adipocyte, chondrocyte, osteoblast) differentiation potential by histochemical staining. Results: The proliferation assays demonstrated that HPLs supported ASC proliferation in a concentration dependent manner, reaching levels that exceeded that observed in the presence of 10% FBS. The concentration of 0.75% HPLs was equivalent to 10% FBS when utilized in cell culture media with respect to proliferation, immunophenotype, and CFU-F frequency. When added to osteogenic, adipogenic, and chondrogenic differentiation media, both supplements showed appropriate differentiation by staining. Conclusion: HPLs is an effective substitute for FBS in the culture, expansion and differentiation of human ASCs suitable for pre-clinical studies; however, additional assays and analyses will be necessary to validate HPLs for clinical applications and regulatory approval.

## 1. Introduction

Human adipose-derived stromal/stem cells (ASCs) are derived from culture expanded stromal vascular fraction (SVF) cells isolated by collagenase digestion from adipose tissue harvested by tumescent liposuction or abdominoplasty [1]. The ASCs are multipotent progenitors that can be distinguished based on their surface antigen immunophenotypic profile and their differentiation potential along the adipocyte, chondrocyte, and osteoblast lineage pathways [1,2]. In addition, there is considerable interest in the ability of ASCs to modulate inflammation in vivo via their secretion of cytokines and exosome vesicles containing microRNAs and proteins [3,4,5,6,7,8,9]. Due to the ease of collection, multipotent differentiation, and paracrine function, ASCs are now being applied in clinical settings to regenerate and repair human tissues impacted by biological aging and disease processes [10,11].

In order to use human ASCs to treat disease, it has been necessary to standardize the methodology for ex vivo expansion in accordance with Current Good Manufacturing Practice (cGMP). Due to the presence of bovine spongiform encephalopathy (BSE) in many herds worldwide, their remains considerable regulatory concern for potential use of FBS as a cell culture nutrient to introduce xenogeneic material [12,13]. Historically, proliferation of ASCs has largely been performed using growth media supplemented fetal bovine serum. While FBS has been an effective medium for cell culture, it does have several additional disadvantages beyond those relating to the risk of BSE contamination. First, FBS products introduce considerable cost to the manufacturing process relative to human platelet lysate due in part to reduced proliferation rates and the need for extended culture expansion periods [14]. Second, FBS use introduces xenoproteins that bind to isolated ASCs, thereby increasing the risk of immune rejection due to antibody development against surface protein complexes on the transplanted ASCs. Indeed, consistent with this concern, the examination of allogeneic transplantation of ASCs in rat models has detected subsequent FBS-related antibody production [15].

To address the potential shortcomings of FBS as a nutrient for clinical expansion of ASCs, investigators have turned to human blood-derived products as alternatives. For example, Finnish investigators have used human serum successfully to expand autologous ASCs for tissue regeneration of craniofacial defects [16]. Likewise, human platelet lysates have long been used as a potential nutrient for the growth of human cells in vitro [17,18]. Independent investigators have begun to explore the use of human platelet lysates as an FBS substitute in culture medium for human ASC expansion [19,20,21,22,23,24,25,26,27,28,29,30]. To extend this line of research, the current study evaluated a human platelet lysate-derived substitute for FBS based on human ASC proliferation, colony forming unit-fibroblast (CFU-F), and differentiation assays.

## 2. Methods

### 2.1. Materials

All reagents were obtained from Thermo Fisher Scientific (Rochester, NY, USA) or LaCell LLC (New Orleans LA) unless stated otherwise.

### 2.2. Human Platelet Lysate Preparation

Platelet lysate was generated from expired bags of concentrated platelets donated by anonymous consenting donors (n = 3 to 4 donors per lot) obtained from a local blood bank (LifeShare Blood Center, Shreveport & Baton Rouge, LA). The platelets were stored on dry ice during transport. Platelets underwent three rounds of freezing (−80 °C overnight) and thawing (18–24 h at 4–8 °C) and were subsequently transferred to sterile centrifugation tubes (Thermo Scientific, Rochester, NY, USA) within a BSL2 cabinet (Class II A/B3 Biological Safety Cabinet, Thermo Forma, USA). In order to remove particulates, the samples were centrifuged at 4000 rpm for 15 min (Sorval Legend T, Kendro, Germany) and the supernatant was aspirated above the pelleted material. The aspirated platelet lysate solution from n = 3 individual donors was pooled, 0.22 μM sterile filtered, and stored at −20 °C prior to use.

### 2.3. Adipose Derived Stromal/Stem Cells

Primary human ASCs were isolated from the lipoaspirate of multiple anonymous healthy female donors (n = 7) with a mean body mass index (BMI) of 25.77 ± 2.82 (± standard deviation) and a mean age of 46.14 ± 14.34 years as previously described (Table 1) [31,32]. All subjects provided informed written consent under a LaCell sponsored protocol reviewed and approved by the Western Institutional Review Board (Pulyallup, WA, USA) as Study Number 1,138,160 and IRB (Institutional Review Board) Tracking Number 20,130,449 with a most recent approval date of March 9, 2019. The donors were either Caucasian (n = 5) or African-American (n = 2). Lipoaspirate was transferred to a sterile 250 mL bottle. The tissue was washed with an equal volume of prewarmed (37 °C) sterile phosphate buffered saline (PBS), and then centrifuged at 1200 rpm for 5 min. The infranatant was aspirated before the tissue was washed and centrifuged again. An equal volume of prewarmed (37 °C) sterile PBS supplemented with type I collagenase (1 mg/mL of tissue) (Worthington Biochemical Corporation, Lakewood, NJ, USA), Fraction V bovine serum albumin (BSA) (10 mg/mL) (Sigma-Aldrich, Saint Louis, MO, USA), and 2 mM CaCl_2_ was added to the tissue. The resulting suspension was then put on a shaker (Innova 4200 Incubator Shaker, New Brunswick Scientific, Edison, NJ, USA) between 180 and 200 rpm at 37 °C for between 50 and 70 min. The digested tissue was then centrifuged at 1200 rpm for five minutes. After gently resuspending the separated tissue, this step was repeated. The supernatant was aspirated and the cell pellet, the stromal vascular fraction (SVF), was transferred to a sterile centrifugation tube and resuspended in prewarmed (37 °C) sterile PBS. The resulting solution was centrifuged at 1200 rpm for five minutes. The PBS was aspirated before the cell pellet was resuspended in LaCell StromaQual™ media (LaCell, New Orleans, LA, USA) containing 10% FBS and plated in T150 or T175 flasks at a density of 0.19 to 0.22 mL of digested lipoaspirate per cm^2^ and incubated in a humidified 5% CO_2_ incubator (Heratherm^®^ microbiological incubators, Thermo Scientific, Logan, UT, USA) for 24 to 48 h. The media was vacuum aspirated, and the flask was washed with prewarmed (37 °C) sterile PBS, fed with 35 mL of fresh stromal media, and the flask returned to the incubator. Upon reaching 80% to 90% confluence, the adherent cell layer, composed of ASCs, was detached using 0.05% trypsin/EDTA (ethylenediamine tetraacetic acid). ASCs were resuspended and stored at a density of 1.0 × 10^6^/mL in cryopreservation media (LaCell, LA, USA) in 2.0 mL cryogenic vials in a liquid nitrogen tank (LS 3000 Lab Systems Taylor Warton, Minnetonka, MN, USA) until use. All subsequent studies were performed with cryopreserved and thawed ASCs used between passages 1 to 3.

### 2.4. Proliferation Assay

ASCs (n = 7) were seeded in stromal media containing 10% FBS, 1.0% PL (Platelet Lysate), 0.75% PL (n = 4), 0.33% PL, or 0.1% PL in 12 well plates (Olympus Plastic, Genesee Scientific, San Diego, CA, USA) at a density of 10,000 cells/cm^2^. Every 24 h for four days, one well of ASCs from each media condition was detached using 0.05% trypsin/EDTA, stained using trypan blue, and counted using a hemocytometer counting chamber with a phase contrast microscope (Motic Microscope, Hong Kong, China).

### 2.5. Colony Forming Unit Assay

ASCs (n = 7) were seeded in six well plates (Olympus Plastic, Genesee Scientific, San Diego, CA, USA) containing LaCell StromaQual™ media supplemented with 10% FBS or 0.75% PL at densities of 100, 200, and 400 cells per well. ASCs were proliferated for two weeks, with the media being changed each week. Colonies were stained with toluidine blue and counted using a phase-contrast microscope. Colonies containing 32 or more cells were counted. Linear regression was performed on the counted colonies, and data are reported as colonies per 100 cells seeded.

### 2.6. Flow Cytometry Assay

Cryopreserved ASCs isolated from n = 4 donors with mean ± S.D. ages of 46.5 ± 7.8 years and BMI of 27.50 ± 3.89 were thawed, cultured in StromaQual supplemented with either 10% FBS or 0.75% PL until confluent, harvested by trypsin digestion, stained with fluorochrome conjugated antibodies (anti-CD29, anti-CD105, anti-CD45, anti-CD34, anti-CD31, anti-CD73, anti-CD90, and isotype control IgG1 (Immunoglobulin G1)), and evaluated by flow cytometry (FACSAria instrument, BD Biosciences, San Jose, CA, USA) as previously described [31].

### 2.7. Differentiation Capacity of ASCs

Confluent ASCs were cultured with adipogenic, osteogenic, as well as stromal media as a control. Passage 1 ASCs (n = 4) were cultured with LaCell StromaQual media containing 10% FBS in T-25 flasks until fully confluent. ASCs were washed with PBS, resuspended in stromal media containing either 10% FBS or 0.75% PL, and seeded on a 12 well plate at a density of 40,000 cells/cm^2^. Once fully confluent, the stromal media was aspirated, and adipogenic and osteogenic differentiation media was introduced.

Pellet ASCs were cultured with chondrogenic containing 10% FBS or 0.75% PL, as well as stromal media with the same concentrations of nutrient supplements as a control. Passage 1 ASCs were (n = 3) cultured with LaCell StromaQual media containing 10% FBS in T-175 flasks until fully confluent. ASCs were washed with PBS, and resuspended in 0.5mL of chondrogenic differentiation media at a density of 500,000 cells/cm^2^ in 15 mL conical tubes. The ASCs were centrifuged at 300 G at 22 °C for 5 min to form a pellet at the bottom of the tube. The tops of the conical tubes were loosened to facilitate gas exchange and the pellets were incubated at 37 °C and 5% CO_2_ overnight. Five pellets were then aggregated into 50 mL conical tubes.

### 2.8. Adipogenic Induction

Confluent ASCs were exposed to adipogenic differentiation media (AdipoQual™ LaCell, LA, USA) containing either 3% FBS or 0.75% PL for three days as previously described [32,33]. Adipogenic maintenance media containing the same concentrations of FBS or PL was then introduced, and the cells were maintained for an additional five to six more days, with the media being changed every two to three days. Differentiated cells were fixed by aspirating the differentiation media, washing three times with PBS, adding PBS containing 10% formalin (Thermo Scientific, Boston, UT, USA), and then placed at 4 °C for one hour. The PBS containing 10% formalin was aspirated and 0.22 μm sterile filtered 5% Oil Red O (Sigma-Aldrich, Saint Louis, MO, USA) in isopropanol was added for 15 min at room temperature. The stain was removed, and the sample rinsed three times or more with distilled water until completely clear. Images were captured using Motic Images Plus 2.0 software (Motic, Hong Kong, China) and a phase contrast microscope.

### 2.9. Osteogenic Induction

Confluent ASCs were exposed to osteogenic differentiation media (OsteoQual™, LaCell, LA, USA) containing either 10% FBS or 0.75% PL for eight to nine days, with the media being changed every two to three days according to a modification of previously described methods [31,32,33,34]. Differentiated cells were fixed by aspirating the differentiation media, washing three times with 150 mM NaCl, adding ice cold 70% ethanol (Sigma-Aldrich, Saint Louis, MO, USA), and then placed at 4 °C for one hour. The 70% ethanol was aspirated, and the sample washed three times with distilled water. Then, 0.22 μm sterile filtered 2% Alizarin Red (Sigma-Aldrich, Saint Louis, MO, USA) stain was added for 10 min at room temperature. The stain was removed, and the sample rinsed five times or more with distilled water until completely clear. Images were captured using Motic Images Plus 2.0 software and a phase contrast microscope.

### 2.10. Chondrogenic Induction

ASC pellets were exposed to complete chondrogenic differentiation media (ChondroQual™, LaCell, LA, USA) containing either 10% FBS or 0.75% PL for 14 days in pellet cultures prepared with 0.25 × 10^6^ ASCs, with media being changed every other day according to a modification of previously described methods [35]. As controls, equivalent pellets were maintained in StromaQual medium over the same time period. Differentiated and undifferentiated cell pellets were fixed by aspirating the media, and washing once with PBS. The pellets were fixed in 4% paraformaldehyde solution, paraffin embedded, and then sectioned and stained with 1% Alcian Blue solution (pH 1.0) for 30 min at room temperature (Scytek Laboratories, Logan, UT, USA).

### 2.11. Quantitative Reverse Transcriptase Polymerase Chain Reaction (qRT-PCR)

ASC pellet cultured under the control or chondrogenic inductive conditions with either 10% FBS or 0.75% PL medium were frozen at –80 °C prior to isolation of total RNA using the RNeasy^®^ Mini Kit (Qiagen, Valencai, CA, USA) according to the manufacturer’s instructions. The resulting total RNA (1 µg) was reverse transcribed using the iScript™ cDNA Synthesis Kit (BioRad, Hercules, CA, USA) according to the manufacturer’s instructions. Real time PCR was performed using a CFX96 Touch™ Real Time PCR Detection System (BioRad, Hercules, CA, USA) as follows: 1 cycle at 95 °C for 4 min, 40 cycles of 95 °C for 15 s followed by 60 °C for 1 min, followed by a melt curve of 55 to 95 °C with an increment of 0.5 °C. The amplification was performed in a 20 µL volume containing iQ™ SYBR Green Supermix (BioRad, Hercules, CA, USA) 2× concentrate (10 µL), each of two primers (4 µL from a 1 µM stock for a 200 nM final concentration), and cDNA template (25 ng). The following human primer sets were synthesized by Integrated DNA Technologies (Coralville, IA, USA) (Gene Bank Accession numbers are presented in parentheses):

Aggrecan Forward AAGTATCATCAGTCCCAGAATCTAGCA (NM_001135).

Aggrecan Reverse CGTGGAATGCAGAGGTGGTT.

Collagen I Forward CACCAATCACCTGCGTACAGAA (NM_000088).

Collagen I Reverse ACAGATCACGTCATCGCACAAC.

Collagen II Forward GGCAATAGCAGGTTCACGTACA (NM_001844).

Collagen II Reverse CGATAACAGTCTTGCCCCACTT.

GAPDH Forward TAAAAGCAGCCCTGGTGACC (NM_002046).

GAPDH Reverse CCACATCGCTCAGACACCAT.

Matrilin I Forward AGGGACTGCGTTTGCATTTTT (NM_002379).

Matrilin I Reverse TCAGTAAAGAAATTCACAGCACTCAGA.

The relative expression of each PCR product was normalized relative to the GAPDH (GlycerAldehyde 3 Phosphate DeHydrogenase) as a control.

### 2.12. Statistical Analysis

All values are reported as mean ± standard error. The student’s T-test was performed on equivalent experiments between PL and FBS, with significance being defined as results with a *p* value < 0.05.

## 3. Results

### 3.1. Effect of Platelet Lysate Concentration in Culture Medium on ASC Proliferation

**Figure 1:**
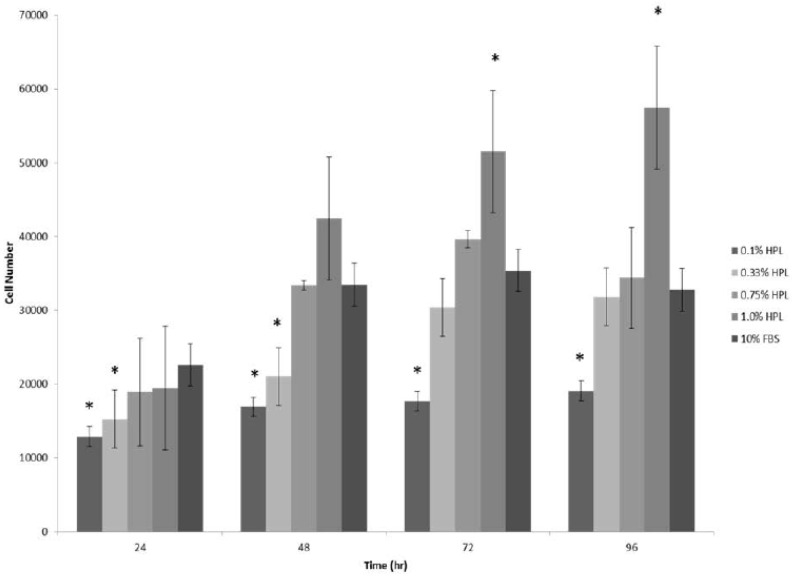
Effect of the concentration of HPL (Human Platelet Lysate) supplementation on ASC proliferation compared to 10% FBS. Stromal media containing 0.1%, 0.33%, 0.75%, 1.0% PL, and 10% FBS were tested for impact on ASC proliferation. Data is reported as the mean ± standard error. * Significant difference; *p* < 0.05.

Initial studies evaluated the impact of platelet lysate concentration on ASC proliferation in vitro. ASC cultures were initiated in stromal media supplemented with increasing concentrations of HPLs (Figure 1). These displayed a concentration dependent change in cellular proliferation when compared to equivalent media prepared with 10% FBS. The FBS used in the study was lot characterized based on its reproducible ability to support robust human ASC proliferation and adipogenesis in vitro. An equivalent number of ASCs were plated in each experiment. Data indicated that increasing the concentration of HPL provided a growth advantage relative to 10% FBS at the 72 and 96 h time points. After comparing ASC proliferation in 0.1%, 0.33%, and 1.0% HPL versus 10% FBS, the experiment was repeated with 0.75% HPL.

### 3.2. ASC Surface Immunophenotype as a Function of Medium Composition

**Figure 2:**
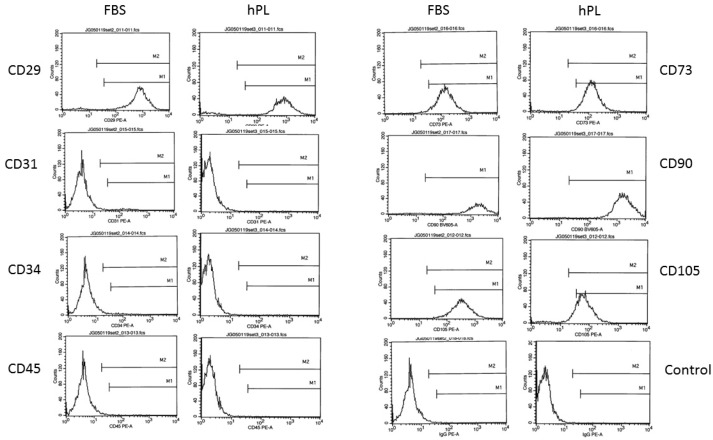
Histograms of flow cytometry detection of surface antigens. The ASCs were culture expanded in stromal media containing either 10% FBS or 0.75% PL and the expression determined for the following surface antigens by flow cytometry: CD29, CD31, CD34, CD45, CD73, CD90, and CD105, with IgG serving as a negative control. The histograms displayed are all derived from a single individual ASC donor and are representative of n = 3 donors. CD: cluster of differentiation.

Successive studies evaluated the impact of 0.75% human platelet lysate as compared to 10% FBS supplementation on the surface immunophenotype of ASCs cultured in stromal medium. Flow cytometry analyses were performed focusing on phenotypic ASC surface antigens as recommended by the International Society for Cell Therapy (ISCT) and International Federation for Adipose Therapeutics and Science (IFATS) consensus [1]. The outcomes indicated that the ASCs displayed comparable levels of characteristic surface antigens CD29, CD31, CD34, CD45, CD70, CD90, and CD105 based on the percentage of positive staining cells independent of the nutrient supplement (Figure 2 and Table 2; mean of n = 4 ASC donors).

### 3.3. Colony Forming as a Function of Medium Composition

**Figure 3:**
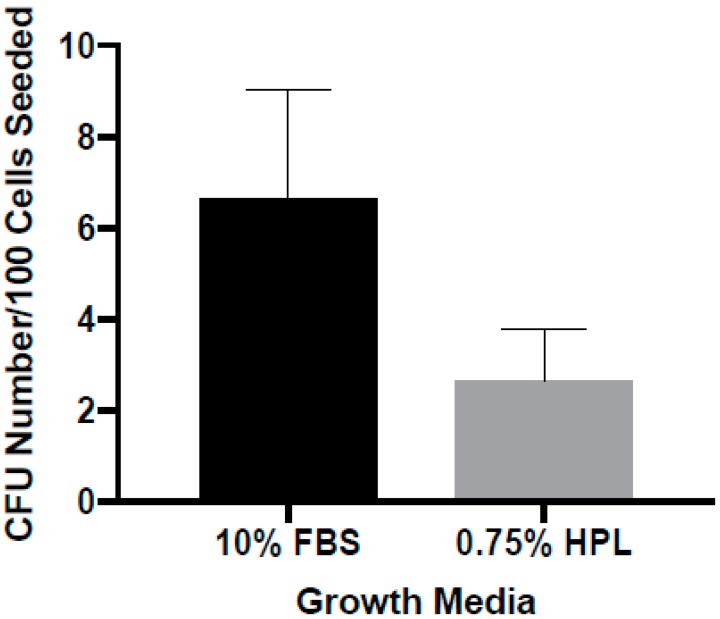
Effect of 0.75% HPL supplementation on the colony-forming unit-fibroblast assay compared to 10% FBS per 10^2^ ASC. Stromal media containing 0.75% PL and 10% FBS were tested for impact on ASC colony-forming unit-fibroblast count. Data are reported as the mean ± standard error.; n = 7, one replicate.

A colony-forming unit-fibroblast assay was performed to assess ASCs’ ability to form colonies in stromal media supplemented with 0.75% PL compared to 10% FBS at passage 1 (Figure 3). The observed morphology of colonies grown in both media was essentially equal, with representative images shown in Figure 3. There was no statistically significant difference in CFU-F numbers as a function of nutrient supplement (10% FBS: 6.65 ± 2.38, 0.75% HPL: 2.56 ± 1.15 colonies, n = 7, *p* value = 0.11).

### 3.4. Differentiation as a Function of Medium Nutrient Supplement Composition

**Figure 4:**
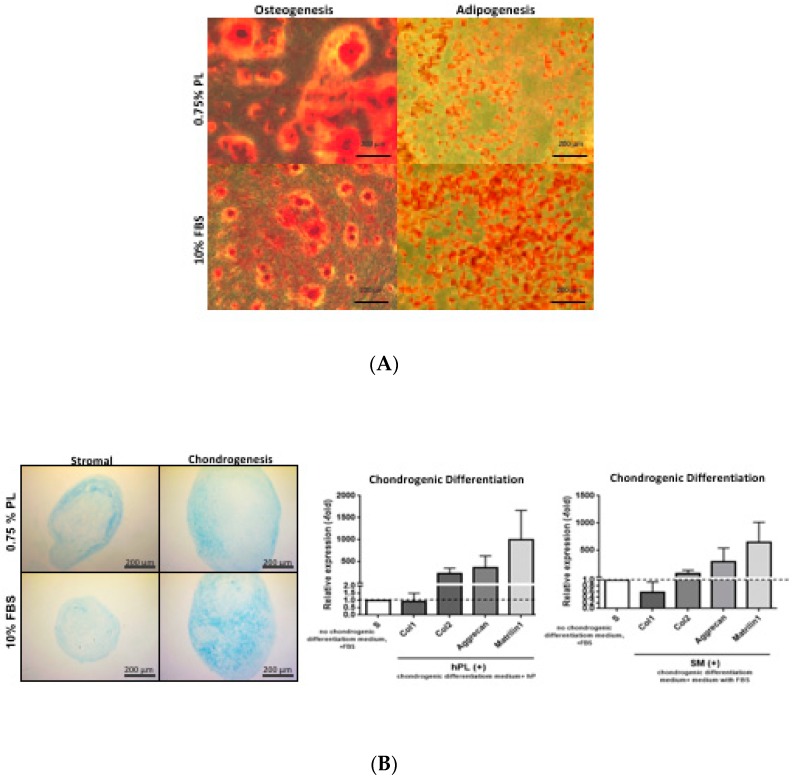
Adipogenic, chondrogenic, and osteogenic differentiation ability of ASCs cultured in 0.75% HPL or 10% FBS containing media. (**A**) Differentiation of ASCs confirmed by staining of two-dimensional cultures with Oil Red O (adipogenesis in the presence of AdipoQual) or Alizarin Red (osteogenesis in the presence of OsteoQual). (**B**) Differentiation of ASCs confirmed by staining with Alcian Blue (chondrogenesis in the presence of ChondroQual vs. StromaQual controls) and by qRT-PCR analysis of the transcribed total RNA for the mRNAs collagen I (Col I), collagen II (Col II), aggrecan, and matrilin 1 (qRT-PCR reactions were conducted in triplicate).

Characterization of ASCs’ ability to differentiate along mesenchymal lineages, specifically adipogenic, osteogenic, and chondrogenic, in differentiation media containing 0.75% PL compared to 10% FBS was qualitatively assessed using histochemical staining (Figure 4A,B). Adipogenesis was assessed based on Oil Red O staining of the intracellular neutral lipid droplets. Adipogenesis was observed in ASCs cultured in AdipoQual prepared with either 0.75% HPL or 10% FBS differentiation media. While both nutrient supplements supported differentiation, adipogenesis was more robust in the presence of FBS. Osteogenesis was assessed based on alizarin red staining of extracellular calcium phosphate deposition and mineralization. While OsteoQual prepared with both nutrient supplements supported differentiation, stain uptake was greater in the presence of 0.75% HPL. Chondrogenesis was assessed based on alcian blue staining of glycosaminoglycan deposition in 3-dimensional pellet cultures. ChondroQual prepared with either nutrient supplement supported chondrocyte formation based on histochemical detection of glycosaminoglycan. While the presence of ChondroQual appeared to increase the relative diameter of the pellet cultures relative to StromaQual, the actual percentage increase was not quantified. Additionally, ChondroQual prepared with either 0.75% HPL or 10% FBS induced a consistent qRT-PCR fold-expression profile for the chondrogenic associated mRNAs aggrecan, collagen type II, and matrilin 1 relative to StromaQual control medium. In contrast, the expression of collagen I was comparable regardless of whether pellets were cultured in the presence of medium with or without chondrogenic inductive agents.

## 4. Discussion

The current study validates HPL as a potential nutrient substitute for FBS in the culture and differentiation of human ASCs. The HPL over a range of concentrations promotes enhanced ASC proliferation in a dose dependent manner. Indeed, the ASC proliferation in the presence of 0.75% HPL is approximately equal to that observed in the presence of 10% FBS. Nevertheless, the two supplements are not identical with respect to all outcomes. While the number of CFU-F tends to be greater in the presence of FBS compared to HPL, this did not reach statistical significance. Based on histochemical analyses, the ASC are capable of undergoing adipogenic, chondrogenic, and osteogenic differentiation in the presence of either FBS or HPL nutrient supplementation. This was further supported by qRT-PCR analysis of a select panel of chondrogenic mRNA biomarkers, which were induced to a comparable fold-expression level independent of the presence of either FBS or HPL. Nevertheless, further quantitative analyses of histochemical stain elution and more comprehensive qRT-PCR analysis of lineage specific mRNAs will be necessary to determine if the relative level of differentiation along any one lineage is favored by one or the other nutrient supplement.

These outcomes confirm findings reported by a growing body of literature. Platelet lysate was initially used as a cell culture supplement providing growth factors to promote CFU formation from breast cancers [17,18]. Likewise, the current study demonstrates that HPL supports CFU-F from ASCs in a manner comparable to FBS, consistent with prior observations reported by Chowela et al. [29]. Additional studies have further examined the utility of platelet lysate as an FBS substitute for the growth of bone marrow-derived mesenchymal stem/stromal cells (BM-MSCs) and ASCs [19,20,21,22,23,24,26,27,28,29,36,37,38]. Consistent with the current findings, HPL displayed support of ASC and BM-MSC proliferation in a concentration dependent manner [19,20,23,24,27,28,30,36,39]. Further analyses correlated the proliferative effects of HPL to the enriched presence of cytokines, including Acrp30 (Adiponectin), bFGF (basic Fibroblast Growth Factor), IL-6, MCP-1 (Monocyte Chemoattractant Protein-1), and PDGF, relative to FBS [39]. Additionally, mass spectroscopic analyses have determined that HPLs contains abundant levels of actin, fibrinogen, tropomyosin, and tubulin [36]. Both Cholewa et al. and Naaijkens et al. noted that HPLs significantly promoted ASC proliferation relative to FBS based on an increased number of population doublings and doubling times, respectively [26,29]. Additionally, they and Blande et al. noted that the size of individual ASCs was larger when cultured in FBS as compared to HPL [19,26,29]. Such observations are consistent with a recent cost analysis, which concluded that HPL is substantially more economical than FBS for clinical grade ASC expansion due to accelerated growth rates [14]. Comparable to the current analysis, Blande et al., Cholewa et al., and Naaijikens et al. independently demonstrated that the immunophenotype of ASCs cultured in FBS and HPL is comparable; however, Naaijikens et al. alone reported an increased intensity of the CD73, CD90, and CD166 surface antigens in the presence of HPL as compared to FBS [19,26,29]. Likewise, these same groups demonstrated that the ASCs cultured in either FBS or HPL continued to display adipogenic, chondrogenic, and/or osteogenic differentiation potential [19,26,29]. Based on this background, groups have begun adapting HPL supplements in the large scale production of human ASCs suitable for clinical applications [22,23].

While there are substantial advantages to the substitution of expired human platelet lysates for FBS in manufacturing protocols for clinical grade ASCs, there are potential issues remaining to be addressed. First is the question of how strong the supply chain will be for expired platelets from authorized blood centers. There is a need for careful consideration of the supply and demand for human platelets. It remains to be determined if an expanded outreach to the blood donor community will be necessary to ensure an appropriate balance between supply and demand. Second is a better understanding of the characterization of HPL expanded ASCs with respect to a wider range of clinical translational applications. Further studies will be necessary to evaluate the HPL expanded ASC product with respect to immunogenicity, immunosuppression, and exosome/secretome expression. Each of these outcomes may influence the utility of ASCs in the context of organ transplantation, immune regulation, and acute and chronic disease therapies [4,10,11]. Nevertheless, the current work and existing literature demonstrate the feasibility of substituting HPL for FBS as an alternative cell culture nutrient supplement and its practicality for in vitro and pre-clinical discovery research.

## Figures and Tables

**Table 1:** Adipose derived stromal/stem cell donor demographic information.

Donor	Race	Age	BMI
L111110W	AA	55	24.56
L110411W	C	66	25.28
L110822W	C	56	26.62
L100401T	C	41	23.73
L100723W	C	22	23.59
L100910W	C	44	24.89
L145	AA	39	31.75
Average		46.14 ± 14.35	25.77 ± 2.82

**Table 2:** Immunophenotype of adipose derived stromal/stem cells following expansion in 10% FBS or 0.75% HPL.

Antibody	FBS	HPL
CD29 PE-A	93.97 ± 3.56	81.80 ± 29.50
CD105 PE-A	95.66 ± 3.66	90.47 ± 10.52
CD45 PE-A	–0.1 ± 0.82	0.02 ± 0.37
CD34 PE-A	2.98 ± 2.35	0.89 ± 0.47
CD31 PE-A	0.53 ± 1.19	0.23 ± 0.27
CD73 PE-A	93.69 ± 5.10	92.74 ± 6.44
CD90 BV605-A	93.66 ± 3.08	99.15 ± 0.75
IgG PE-A	0.32 ± 0.19	−0.10 ± 0.37
PE-A	0.74 ± 0.79	0.33 ± 0.65
